# A rare case of a spontaneously ruptured secondary hepatic malignant lymphoma

**DOI:** 10.1186/s40792-018-0451-2

**Published:** 2018-05-03

**Authors:** Ko Oshita, Toshiyuki Itamoto, Akihiko Oshita, Hideki Nakahara, Takashi Nishisaka

**Affiliations:** 1 Department of Gastroenterological Surgery, Hiroshima Prefectural Hospital, 1-5-54 Ujina-kanda, Minami-ku, Hiroshima, Japan; 2 Department of Pathology Clinical Laboratory, Hiroshima Prefectural Hospital, Hiroshima, Japan; 3 Department of Gastroenterological and Transplant Surgery, Applied Life Sciences, Institute of Biomedical and Health Sciences, Hiroshima University, Hiroshima, Japan

**Keywords:** Hepatic malignant lymphoma, Spontaneous rupture, Hemoperitoneum, Transcatheter arterial embolization, Hepatectomy

## Abstract

**Background:**

Although secondary liver involvement of the lymphoma is common and occurs in 50% of patients with non-Hodgkin’s lymphoma, liver tumor rupture in malignant lymphoma is extremely rare. We report a case of a spontaneously ruptured secondary liver involvement of non-Hodgkin’s lymphoma that was successfully treated with transcatheter arterial embolization (TAE) to obtain hemostasis, and subsequent hepatectomy and systemic chemotherapy. To the best of our knowledge, this is only the second reported case of a ruptured hepatic lymphoma.

**Case presentation:**

A 74-year-old man with sudden-onset right shoulder and upper quadrant pain was transferred to our hospital via an ambulance. His vital signs were stable. Contrast-enhanced computed tomography showed a large hypo-enhancing tumor (94 × 81 mm) in the posterior segment of the liver, with intratumoral extravasation and a 12 × 10 mm daughter tumor in segment 5 of the liver. Hemoperitoneum due to rupture of hepatocellular carcinoma with intratumoral hemorrhage was suspected, although the serum alpha-fetoprotein and protein induced by vitamin K absence-II levels were within normal range. TAE was used for hemostasis. Extended posterior segmentectomy including tumor resection in segment 5 was performed on day 23 after embolization. The postoperative course was uneventful. Pathological examination of the resected specimens revealed that the ruptured tumor was diffuse large B-cell lymphoma. Postoperative fluorodeoxyglucose positron emission tomography-computed tomography showed uptake in the left parotid gland, pancreas, and thoracic vertebra. Based on these findings, the final diagnosis was a ruptured secondary hepatic malignant lymphoma. Complete remission was achieved with chemotherapy. He remains alive 30 months after hepatectomy without evidence of relapse.

**Conclusions:**

We report the first case describing a hepatic tumor rupture as the first presentation of a primary or secondary hepatic malignant lymphoma. The patient was successfully treated with TAE, hepatectomy, and subsequent systemic chemotherapy for non-Hodgkin’s lymphoma.

## Background

The liver is an organ that can either be involved in widespread lymphoma or rarely as a primary site of lymphoma. Secondary liver involvement of the lymphoma is common and occurs in 50% of patients with non-Hodgkin’s lymphoma. Moreover, the liver is the third most common abdominal organ with lymphoma involvement, following the spleen and gastrointestinal tract [1, 2]. The most common secondary hepatic lymphoma is non-Hodgkin’s lymphoma [3]. Diffuse large B-cell lymphoma is an aggressive non-Hodgkin’s lymphoma with increasing incidence in elderly patients. However, liver tumor rupture in patients with malignant lymphoma is extremely rare [4, 5].

To the best of our knowledge, this is only the second reported case of a ruptured hepatic lymphoma. Herein, we describe a patient with a spontaneously ruptured secondary liver involvement of non-Hodgkin’s lymphoma successfully treated via transcatheter arterial embolization (TAE) to obtain hemostasis and via subsequent hepatectomy and systemic chemotherapy.

## Case presentation

A 74-year-old man with sudden-onset right shoulder and upper quadrant pain came to our hospital via an ambulance. His medical history included chronic obstructive pulmonary disease and appendectomy. He reported no history of trauma or falls. He had not been prescribed anticoagulants or antiplatelet agents. On physical examination, he was conscious and alert, and his blood pressure was 109/65 mmHg; heart rate, 97 beats/min; and respiratory rate, 20 breaths/min. He complained of mild abdominal distension and tenderness on the right upper quadrant area. Laboratory examination showed white blood cells of 15,700/μL; hemoglobin, 11.3 g/dL, platelet count, 236,000 /µL; aspartate aminotransferase (AST), 57 U/L; alanine amino transferase (ALT), 43 U/L; serum level of lactate dehydrogenase, 454 U/L; alkaline phosphatase, 218 IU/L; and total-bilirubin, 0.5 mg/dL. The results of coagulation tests were normal. Serum alpha-fetoprotein and protein induced by vitamin K absence-II levels were within normal range (5 ng/mL and 26 mAU/mL, respectively). Hepatitis B virus surface antigen and hepatitis C virus antibody were negative.

**Fig. 1:**
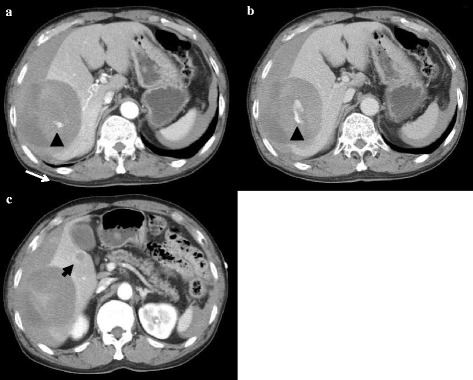
Contrast-enhanced computed tomography scan showed a hypo-enhancing tumor (98 × 81 mm) in the posterior segment of the liver with intratumoral extravasation on arterial phase (**a**) and on delayed phase (**b**). A daughter tumor (12 × 10 mm) in segment 5 of the liver was also detected (**c**)

**Fig. 2:**
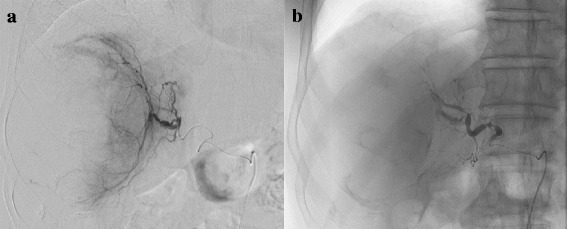
Transcatheter arterial embolization was performed. Selective hepatic angiogram revealed that the branches of the right posterior hepatic artery were stretching with peripheral enhancement of the liver with no extravasation (**a**). The right posterior hepatic artery was embolized with a gelatin sponge (**b**)

Contrast-enhanced computed tomography (CT) showed a large hypo-enhancing tumor (94 × 81 mm) in the posterior segment of the liver with intratumoral extravasation and a 12 × 10 mm daughter tumor in segment 5 (S5) of the liver (Fig. 1a, b). Hemoperitoneum due to rupture of hepatocellular carcinoma (HCC) with intratumoral hemorrhage was suspected, and TAE was selected for obtaining hemostasis. Selective hepatic arteriogram from the right posterior hepatic artery revealed that the arterial branches were stretching with peripheral enhancement of the tumor. No extravasation of contrast medium could be detected during the procedure (Fig. 2a). The right posterior hepatic artery was embolized with a gelatin sponge (Fig. 2b). The serum AST and ALT levels, which were 358 and 327 IU/L respectively, peaked on the second day and improved within the normal range on the 12th day after TAE. The retention rate of indocyanine green at 15 min on the ninth day after TAE was 6.4%.

**Fig. 3:**
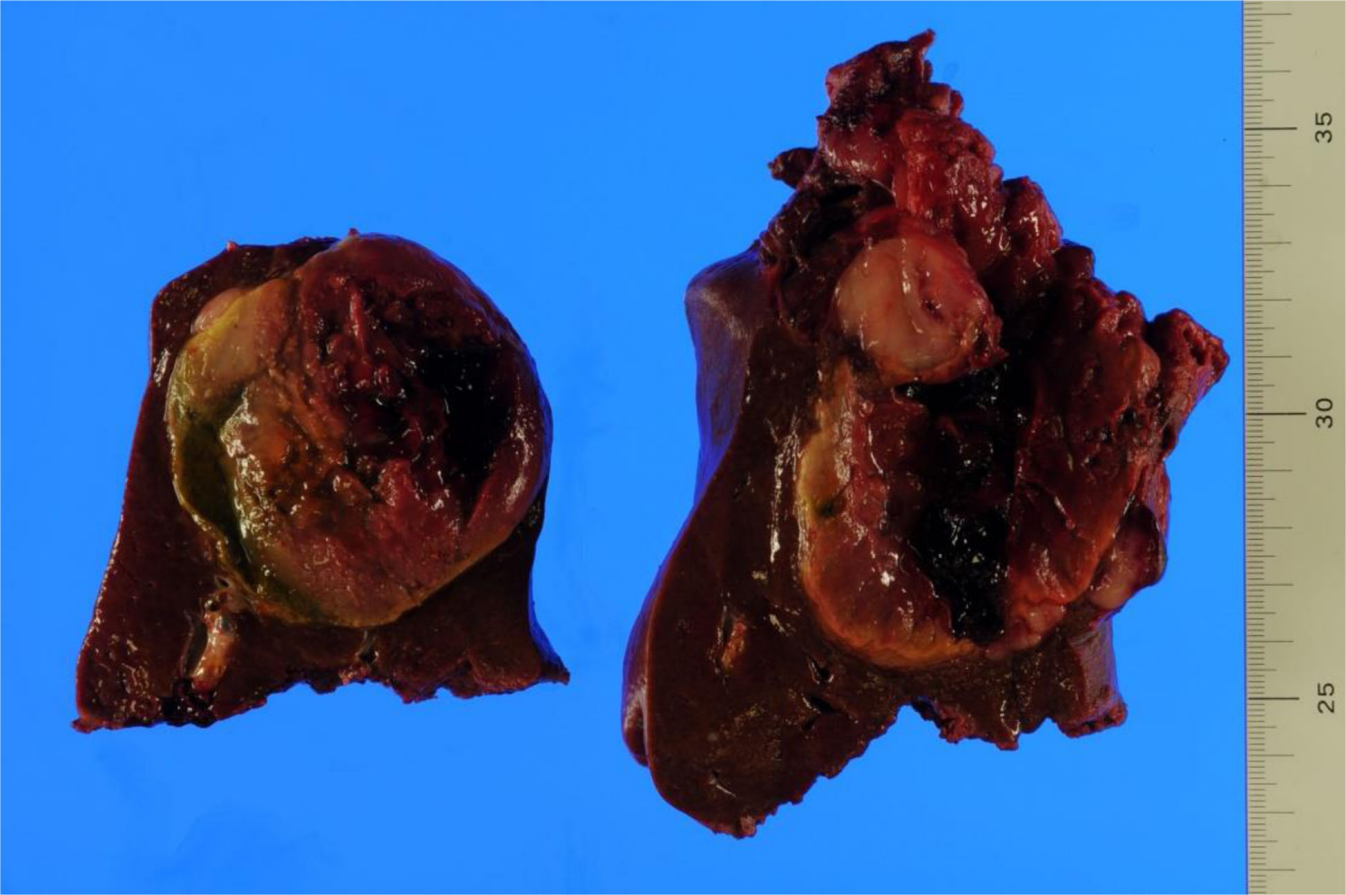
Specimen of the resected posterior segment (S6/7) including a tumor in S5 of the liver. The majority of ruptured tumor was necrotized

Extended posterior segmentectomy (resection of S6 + S7) including removal of a tumor in S5 of the liver was performed on the 23rd day after TAE. Substantial clots and old blood were found in the abdominal cavity. A tumor in the posterior segment adhered to the right diaphragm, and a part of the tumor capsule had been ruptured. Intraoperative echogram and palpation revealed an incidental tumor of 1.2 cm in diameter in S3 of the liver. Additional procedure included partial resection of S3 of the liver. The resected specimen revealed that the ruptured tumor in the posterior segment was 97 × 67 mm in size, and the majority of the tumor was necrotized due to TAE (Fig. 3).

**Fig. 4:**
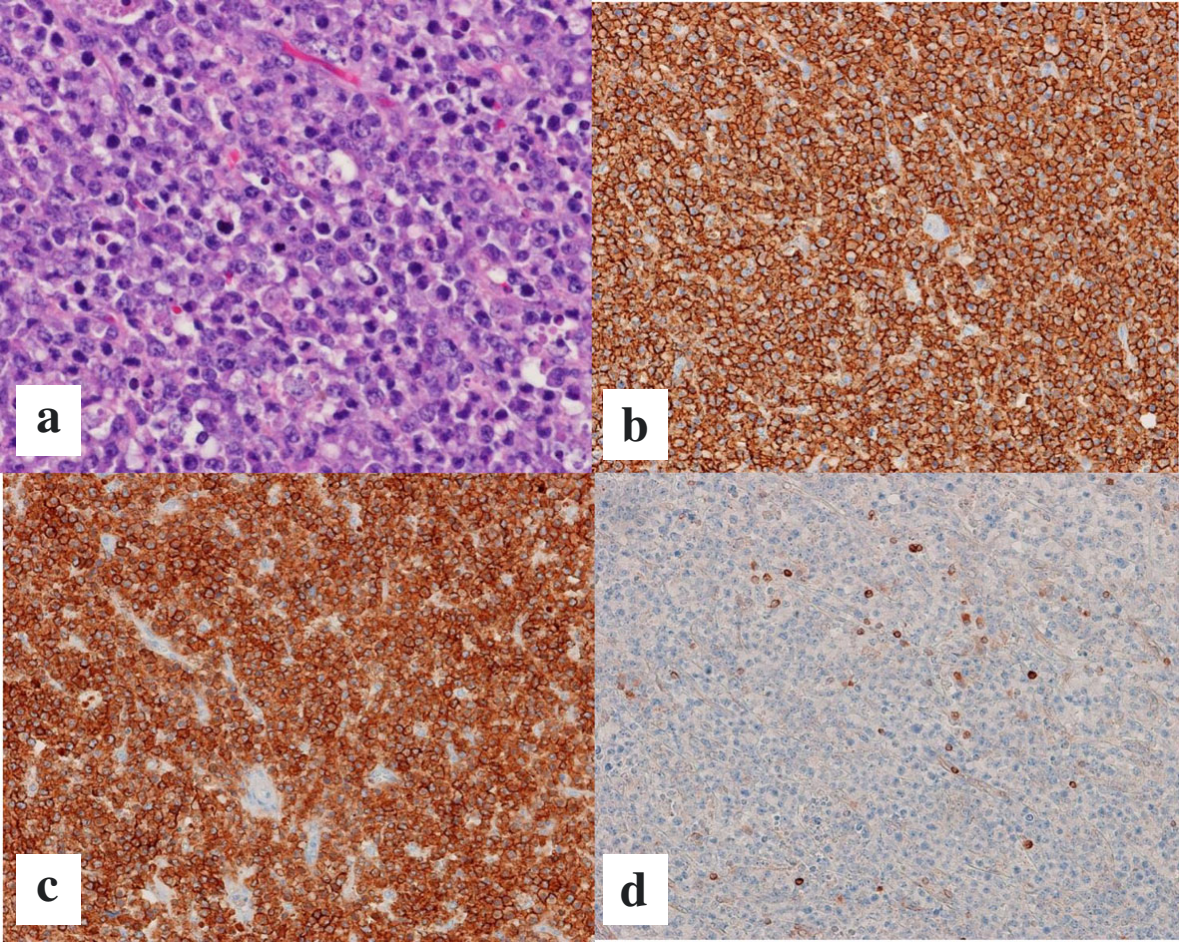
Hematoxylin and eosin staining (× 200 magnification) demonstrated that viable lesions in the tumor were composed of diffusely proliferated atypical lymphoid cells (**a**). Immunohistochemical staining (× 200 magnification) revealed that the tumor cells were diffusely positive for CD20 (**b**) and CD79a (**c**) and focally positive for Bcl-2 (**d**)

**Fig. 5:**
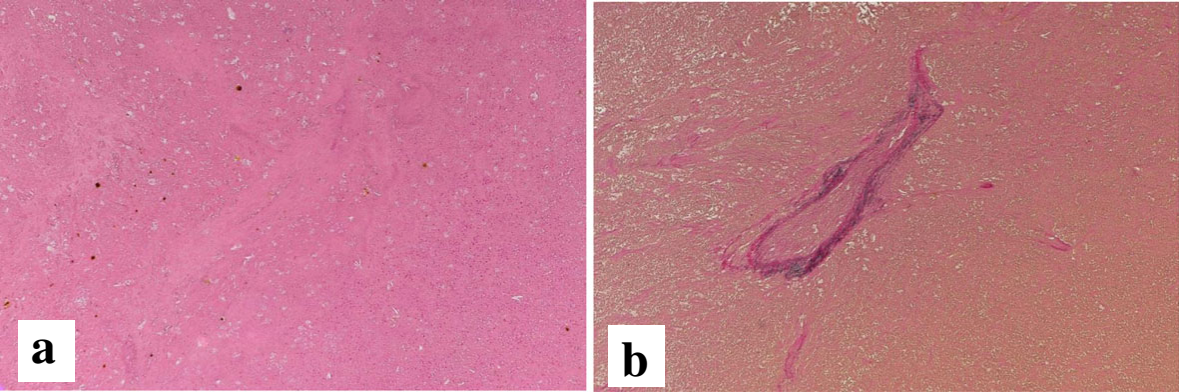
Hematoxylin and eosin staining (× 40 magnification) showed massive coagulation in the central part of the tumor (**a**). Verhoeff-Van Gieson staining (× 40 magnification) of the area revealed a relatively large artery penetrating the necrotic tissue (**b**)

Histologic examinations revealed that viable lesions were composed of diffusely proliferated atypical lymphoid cells (Fig. 4a). Immunohistochemical staining of these tumor cells was negative for hepatocyte marker, diffusely positive for CD20 and CD79a, and focally positive for Bcl-2 (Fig. 4b–d). Histological examination also revealed a massive coagulative necrosis in the central part of the tumor (Fig. 5a). The Verhoeff-Van Gieson staining of the area revealed a relatively large artery penetrating the necrotic tissue (Fig. 5b). Approximately 90% of the tumor cells were positive for Ki-67. These findings confirmed non-Hodgkin’s diffuse large B-cell lymphoma.

The serum interleukin-2 receptor level measured after identification of the pathological findings from the resected specimens was 893 U/L. The postoperative course was uneventful, and he was discharged from the hospital on postoperative day 13.

Fluorodeoxyglucose positron emission tomography-computed tomography (PET-CT) performed 1 month after operation showed an uptake in the left parotid gland, pancreas, remnant liver, and the thoracic vertebrae. For systemic non-Hodgkin’s lymphoma, the patient received eight cycles of rituximab, cyclophosphamide, doxorubicin, vincristine, and prednisolone regimen and achieved complete remission, which has been maintained to date for 30 months.

## Discussion

Rupture of a liver tumor occasionally occurs, and the associated intraperitoneal hemorrhage is potentially life-threatening. The most common symptom of ruptured liver tumor is sudden onset of abdominal pain with shock. HCC is the most common cause of a spontaneous hemorrhage with 10% of these cases presenting with abdominal bleeding [6, 7]. However, hepatic tumor rupture in patients with malignant lymphoma is extremely rare [4, 5].

Tsutani et al. first reported a case of ruptured malignant lymphoma in 1999 [4]. Following initial treatment with chemotherapy and irradiation for non-Hodgkin’s lymphoma (diffuse large B-cell lymphoma) of the anterior chest wall and para-aortic lymph nodes, disease recurrences occurred in the liver and vertebra. When recurrent multifocal liver tumors decreased in size after salvage chemotherapy including irinotecan, the patient died of intraperitoneal bleeding. Autopsy revealed ruptured lymphomas of the liver. It was speculated that marked anti-tumor effect reducing the tumor size might have caused severe necrosis and subsequent rupture of the liver tumors.

Our case presented herein is the second report describing hepatic tumor rupture and hemoperitoneum that occurred in malignant lymphoma. The patient has ruptured hepatic tumor treated successfully via TAE followed by hepatectomy. The clinical diagnosis was HCC rupture. Pathological examination of the resected specimens revealed that the ruptured tumor was non-Hodgkin’s lymphoma. Postoperative PET-CT revealed that the patient had systemic disease of non-Hodgkin’s lymphoma. In non-Hodgkin’s lymphoma, liver involvement is observed in as much as 50% of autopsy cases. However, tumor rupture and hemoperitoneum have never been described as a first presentation of primary or secondary hepatic malignant lymphoma [8, 9]. This is the first report describing such case.

Tsutani et al. suggested that one of the reasons for the low incidence of rupture of malignant lymphoma might be its hypo-vascularity [4]. Majority of hepatic lymphoma lesions demonstrate minimal to no enhancement on all the phases of contrast-enhanced CT. Enhancement, when present, is characteristically less than the surrounding hepatic parenchyma [10, 11]. However, arterial channels can often be seen coursing through the hypo-enhancing tumor [2]. The other pattern on contrast-enhanced CT is target-like appearance, that is, peripheral enhancement with a central non-enhancement, mimicking intrahepatic cholangiocarcinoma or metastatic liver cancer [11, 12]. In our case, ruptured tumor was hypo-enhancing with extravasation of contrast medium within the tumor on contrast-enhanced CT. Although the hypo-vascularity is not likely to be the ruptured HCC, clinical diagnosis of ruptured HCC was made, suggesting that the hypo-vascularity might depend on the intratumoral hematoma due to hemorrhage or spastic changes of the feeding arteries after bleeding.

The mechanism of spontaneous rupture in HCC has been discussed and might most likely involve various factors. Spontaneous rupture tends to occur in large tumors [13], but small lesions located at the surface of liver also bleed and rupture [14, 15]. In the majority of cases, bleeding is probably initiated within the tumor. This leads to a sudden increase in pressure within the tumor followed by bursting through the tumor surface [5, 16]. Bleeding within the tumor can be caused by disruption of a friable feeding artery or by central necrosis in rapidly growing HCC [5, 7, 13]. Central necrosis may lead to disruption of a friable feeding artery [7]. Some investigators have demonstrated impairment of the vascular integrity in patients presenting with spontaneous rupture of HCC [17]. The sudden obstruction of venous drainage caused by rapid tumor growth or tumor thrombus can result in congestion and further necrosis of tumor tissue and can lead to increased intratumoral pressure [14, 16]. Another mechanism of tumor rupture includes laceration of a superficial tumor as a result of repeated respiratory movement, particularly for a tumor located under the diaphragm [16].

In the present rare case of rupture of secondary hepatic lymphoma, systemic malignant lymphoma was diagnosed as a result of hepatic tumor rupture. The cause of the tumor rupture might include bleeding from arterial channels coursing through the hypo-enhancing tumor, which was demonstrated by the existence of intratumoral extravasation of contrast medium on contrast-enhanced CT. The mechanisms of tumor rupture in the present case of malignant lymphoma might be the same as those of HCC rupture resulting from initial bleeding within the tumor due to impairment of the vascular integrity and the subsequent sudden increase in pressure within the tumor followed by bursting through the tumor capsule. Although clinical diagnosis before treatment was ruptured HCC and the treatment strategy applied was similar for HCC, the addition of chemotherapy for malignant lymphoma after surgery contributed to good patient prognosis.

## Conclusions

We report the first case of a hepatic tumor rupture as the first presentation of a primary or secondary hepatic malignant lymphoma. The patient was successfully treated via TAE, hepatectomy, and subsequent systemic chemotherapy for non-Hodgkin’s lymphoma.

## Abbreviations

CT: Computed tomography
FDG: Fluorodeoxyglucose
HCC: Hepatocellular carcinoma
PET-CT: Positron emission tomography-computed tomography
TAE: Transcatheter arterial embolization
